# Clinical Indications of Recombinant Human Erythropoietin in a Single Center: A 10-Year Retrospective Study

**DOI:** 10.3389/fphar.2020.01110

**Published:** 2020-07-24

**Authors:** Xiao-Zhen Guan, Lei-Li Wang, Xue Pan, Li Liu, Xiao-Lin Sun, Xiao-Juan Zhang, De-Qing Wang, Yang Yu

**Affiliations:** ^1^ Department of Transfusion, The First Medical Center of Chinese PLA General Hospital, Beijing, China; ^2^ Center for clinical laboratory Medicine, The First Medical Center of Chinese PLA General Hospital, Beijing, China

**Keywords:** recombinant human erythropoietin, clinical indications, off-label, irregular transfusion, renal failure dialysis, perioperative erythrocyte mobilization, chemotherapy for non-bone marrow malignant tumors

## Abstract

In the 1980s, recombinant human erythropoietin (rhEPO) began to be used in clinical practice. In this study, the clinical application of rhEPO from single-center in recent ten years was reviewed, and the scope of indications and clinical efficacy were evaluated. The medical records of 35829 in-patients who were treated with rhEPO in the first Medical Center of the Chinese PLA General Hospital from 2009 to 2018 were collected. According to the scope of indications approved by CFDA (China Food and Drug Administration), curative effect and off-label of rhEPO were analyzed. Of the 35829 patients, 19013 (53.1%) were male and 16816 (46.9%) were female, with an average age of (52.1 ± 18.6) years. The usage of rhEPO is increasing year by year. The overall effective rate was 53.1%. The number of patients who met the indications accounted for 67.2%, and the effective rate patients with indications and Off-label were 48.8% and 50.7%. Among the patients with irregular use of rhEPO perioperative imperfect laboratory examination patients accounted for the highest proportion (7.1%). The volume of RBC(s) (red blood cell(s)) transfusion in patients with rhEPO was significantly less than that in patients without rhEPO (p<0.05). The use of rhEPO Off-label is very common and has a certain curative effect. It can be used as evidence support for the update of the scope of indications. In addition, There are still irregular use of rhEPO and transfusion in clinic. The unreasonable use of rhEPO and transfusion should be further standardized to ensure the safety and effectiveness.

## Introduction

Erythropoietin (EPO) is an active glycoprotein secreted by the kidney. EPO in blood binds to EPO receptors on the surface of erythroid hematopoietic progenitor cells in bone marrow, which can promote the proliferation and differentiation of erythroid hematopoietic progenitor cells. In the late stage, the maternal RBCs progenitor cells could stimulate the colony formation obviously. At high concentration, it could also stimulate the early maternal RBCs progenitor cells and guide the colony formation (Kuhrt and Wojchowski, 2015). EPO is also an effective apoptosis regulator, which can resist apoptosis by maintaining the stability of mitochondrial membrane, up-regulating the expression of bcl-2 and bcl-xl genes and inhibiting the activation of caspase-3. Therefore, it can delay the apoptosis of erythroid progenitor cells, primitive erythrocytes and a series of hematopoietic cells (Jaspers et al., 2014). The two mechanisms work together to keep the number of mature RBC(s) relatively stable in normal people, increase the number of RBC(s) and the concentration of hemoglobin in anemia patients, and directly improve the oxygen carrying capacity of blood.

Since the discovery of EPO by French scientists Carnot and Deflandre in 1906 (Ribatti, 2008), People have a deep understanding of EPO and have made many great achievements. Using genetic engineering technology, the first rhEPO product of word has been successfully developed and marketed, mainly used in the treatment of anemia caused by renal causes and malignant tumor radiotherapy and chemotherapy. In September 2001, Arnesp, a long-term recombinant lycopene product, was approved by FDA and officially launched in 2002. In November 2007, another long-term recombinant lycopene product, Mircera, was launched. The scope of indications approved by FDA include the treatment of anemia due to cancer chemotherapy (Platanias et al., 1991; Cascinu et al., 1994; Ludwig et al., 1995; Glaspy et al., 1997), zidovudine-treated HIV infection (Fischl et al., 1990; Henry et al., 1992) and elective orthopedic surgery (Group, C. O. P. E. S, 1993; Faris et al., 1996; Goldberg et al., 1996). In china, rhEPO has received Food and Drug Administration (CFDA) approval for the treatment of anemia due to chronic renal failure(Society, 2007), cancer chemotherapy (CSCO, 2010), and elective orthopedic surgery (Company, 2001). A comparison of the differences in indications approved by CFDA and FDA is shown in **Table 1**. Up to now, dozens of rhEPO products have appeared all over the world (Campana, 2007).

**Table 1:** Labeled indication for rhEPO treatment: CFDA versus FDA.

CFDA-approved indications for rhEPO products	FDA-approved indications for rhEPO products
Anemia caused by renal failure(including dialysis and non-dialysis)* Anemia caused by chemotherapy for non-bone marrow malignant tumors^†^ Perioperative erythrocyte mobilization.^‡^	Chronic renal failure* Chemotherapy-treated nonmyeloid malignancy^†^ Elective orthopedic surgery^‡^ Zidovudine-treated HIV infection

*Treatment and maintenance. ^†^Treatment therapy. ^‡^prevention.

In recent years, it has been found that EPO/EPO receptor system is expressed in macrophages, smooth muscle cells, skeletal myoblasts, neuron cells, kidney cells, cardiomyocytes and retinal ganglion cells. The anti-oxidation, anti-apoptosis and anti-inflammatory effects of rhEPO on organs and tissues have been found one after another, and the protective effects of arginine vasopressin (AVP) on various organs have been studied more and more (Rocha et al., 2015; Ji et al., 2016; Liu et al., 2017). rhEPO could enhance the activities of antioxidant enzymes such as superoxide dismutase (SOD), glutathione peroxidase and catalase. Up-regulating the expression of antioxidant enzymes and down-regulating the production of oxygen free radicals play an antioxidant role (Dardashti et al., 2014). EPO can promote nervous system regeneration, inhibit apoptosis, improve cell survival rate, and protect nerve cells(Liu et al., 2006; Zhou et al., 2015; Li et al., 2017). At the same time, it can reduce the release of many kinds of inflammatory factors and reduce the infiltration and injury of inflammatory cells, so as to play the role of organ protection (Liu et al., 2006). Since rhEPO was put into clinical therapy, there has been a lack of big data retrospective investigation on the standardization, efficacy, and indications of clinical drug therapy. Our goal is to conduct a retrospective study on the actual use of clinical rhEPO in the past ten years in China, evaluate the use of rhEPO according to the approved indications and the excess indications, and provide reference basis for the adjustment or expansion of rhEPO indications. Provide evidence-based big data support for clinical rational medication.

## Materials and Methods

### Setting

The study was conducted in the first Medicine Center of the Chinese PLA General Hospital, the largest medical institution in North China. Founded in 1953, the hospital is a large modern comprehensive hospital integrating medical treatment, health care, teaching and scientific research. The number of outpatient emergencies was more than 4.9 million, 198000 people were admitted, and nearly 90 000 cases were operated on each year. As a data research center in the past 10 years as a research interval, the research results should be representative in North China.

### Study Design

#### Included Object

All patients who were treated with rhEPO at the first Medicine Center of the Chinese PLA General Hospital from January 1, 2009 to December 31, 2018.

#### Medication Guide

All the patients included in this study were injected subcutaneously according to the medication specification: Anemia caused by renal failure patients with Hb (hemoglobin) ≤ 120g/L before treatment: 10,000 IU, three times a week; patients with anemia caused by chemotherapy for non-bone marrow malignant tumors whose Hb was 80-120g/L before treatment: 36000IU once a week; For patients with response (Hb rise≥10g/L), if Hb ≥ 120g/L in any case, the use should be stopped. If the patient is responsive to rhEPO and still has anemia symptoms, it should be reevaluated (Society, 2007; CSCO, 2010; Consensus expert Group on diagnosis and treatment of Renal anemia, N. B. o. C. M. A, 2018) Perioperative RBCs mobilization patients: patients undergoing elective surgery with Hb 100-130g/L before operation, 10 days before operation and 4 days after operation, 150IU/kg once a day. The evaluation of curative effect: the increase of Hb≥10g/L after the end of medication was effective (CSCO, 2010). Subsequently, the indications, the advice of doctor, Hb level, Hct (hematocrit) level, application of off-label and unreasonable use of rhEPO were evaluated. The basis includes: clinical practice Guide and Clinical practice recommendation of chronic Kidney Disease anemia in the United States (2006), Chinese expert consensus on diagnosis and treatment of anemia caused by renal failure (2018)(Consensus expert Group on diagnosis and treatment of Renal anemia, N. B. o. C. M. A, 2018), Expert consensus on the rational use of Recombinant Human Erythropoietin in anemia caused by renal failure (2007) (Society, 2007), EPO Clinical practice Guide for the treatment of tumor anemia (2010-2011) (CSCO, 2010).

### Ethics

This study has been approved by Medical Ethics Committee of the General Hospital of the Chinese people’s Liberation Army.(code:S2019-290-01).

### Data Collection

#### Data Sources

The medical records of 35829 inpatients who used rhEPO from January 2009 to December 2018 were collected by the HIS system of the First Medical Center of Chinese PLA General Hospital Drug Source: recombinant human lycopene injection (CHO cell).

#### Variable Collection

General information(patient ID, age, sex, weight, height, weight, diagnosis, department, admission time, discharge time), RhEPO medication information(name, time, dosage, mode of administration), Laboratory examination(RBC(s), Hemoglobin, red blood cell volume, MCV, RDW, MCHC, reticulocyte percentage).

### Statistical Analysis

The measurement data are represented by mean ± standard deviation, and the counting data are expressed by t-test, nonparametric rank sum test and chi-square test. The difference was statistically significant (P<0.05). Stata15.0 version software is used for data analysis.

## Results

### Demographic Characteristics of Patients Using rhEPO

All the 35,829 patients were hospitalized, including 53.1% male and 46.9% female, with an average age of (52.1 ± 18.6) years. The distribution of patients in all age groups is shown in **Table 2**.

**Table 2:** Demographic characteristics of patients using rhEPO in a single center from 2009 to 2018.

Total number of patients taking drugs	35,829
Gender	
Male	19,013 (53.1%)
Female	16,816 (46.9%)
Age	
average age	52.1 ± 18.6
Age division (WHO standard) and proportion (%)	
Minor (0 to 17) years old	1,478 (4.1%)
Young people (18-65)	25,490 (71.1%)
Middle-aged (66-79) Old people (≥80) years old	6,545 (18.3%) 2,316 (6.5%)
Data are reported as number (%).	

### Annual Changes in rhEPO Usage

**Figure 1:**
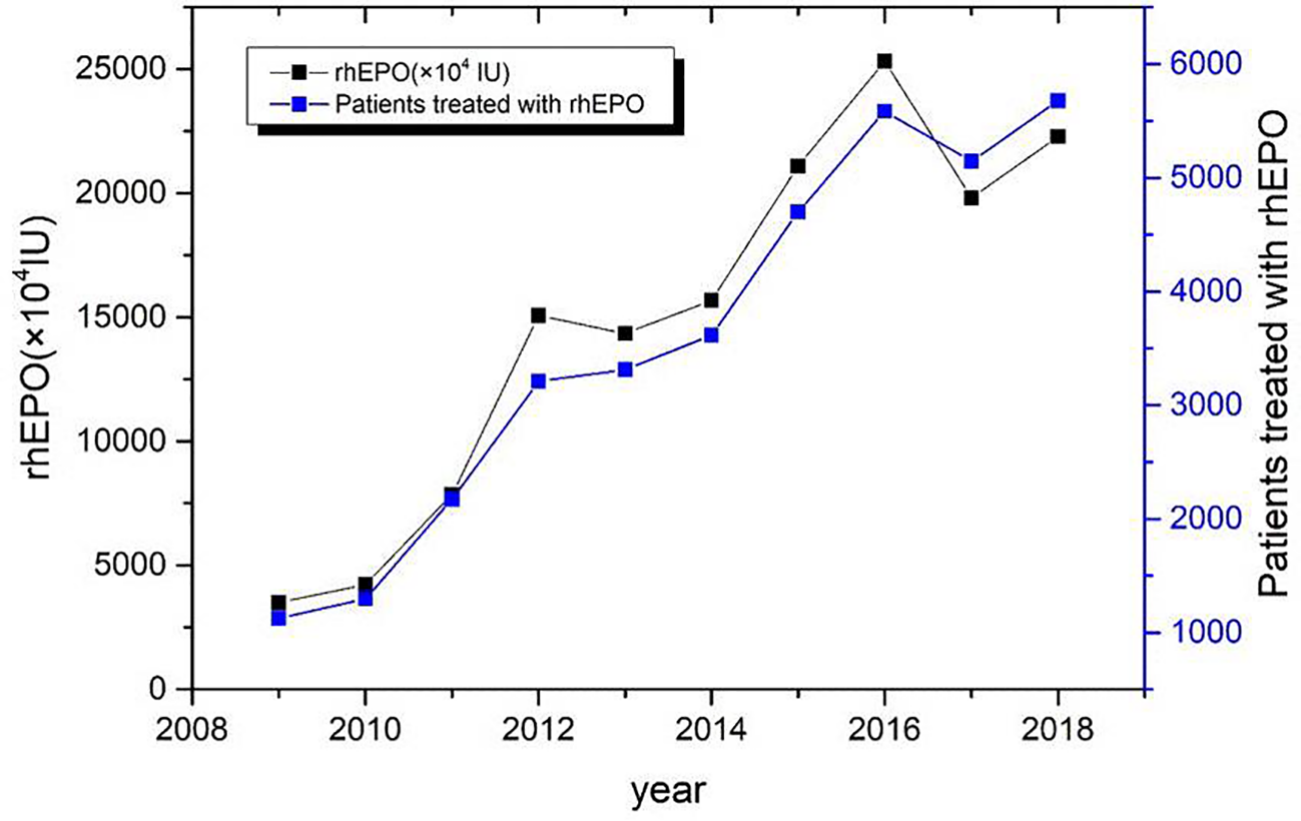
Annual consumption trend chart of rhEPO in a single center from 2009 to 2018.

During the period from 2009 to 2018, the total dosage of rhEPO in 35829 patients was 149170 ×10^4^ IU. The average use of rhEPO per patient was (4.16 ± 4.05) ×10^4^ IU. The trend of clinical use of EPO in the past decade is shown in **Figure 1**.

### Indication Dosage

**Figure 2:**
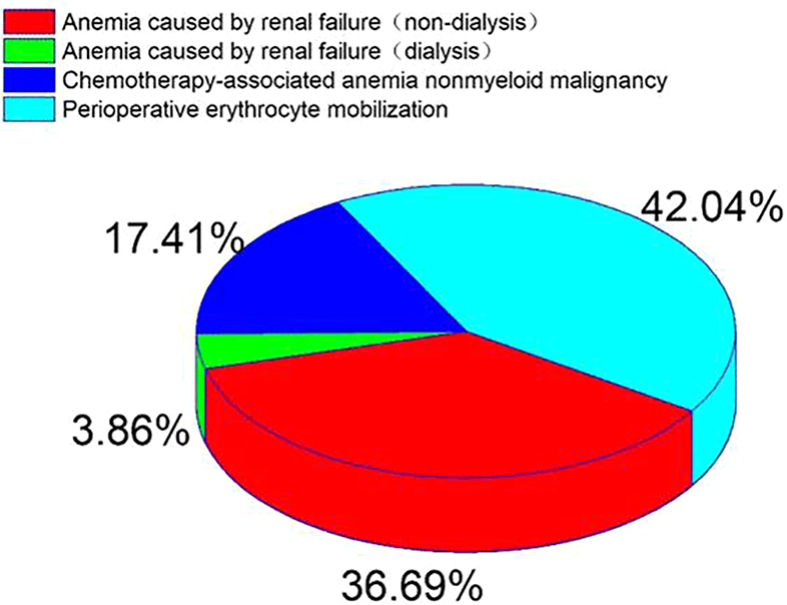
Percentage distribution of diseases in patients with EPO in accordance with indications from 2009 to 2018.

Anemia caused by renal failure, anemia caused by chemotherapy for non-bone marrow malignant tumors and perioperative erythrocyte mobilization accounted for 55.4% of the total dose of 82629 ×10^4^ IU, The average dosage was (3.19 ± 3.0) ×10^4^ IU (**Figure 2**).

### Comparison of the Use of rhEPO in Main Departments

**Figure 3:**
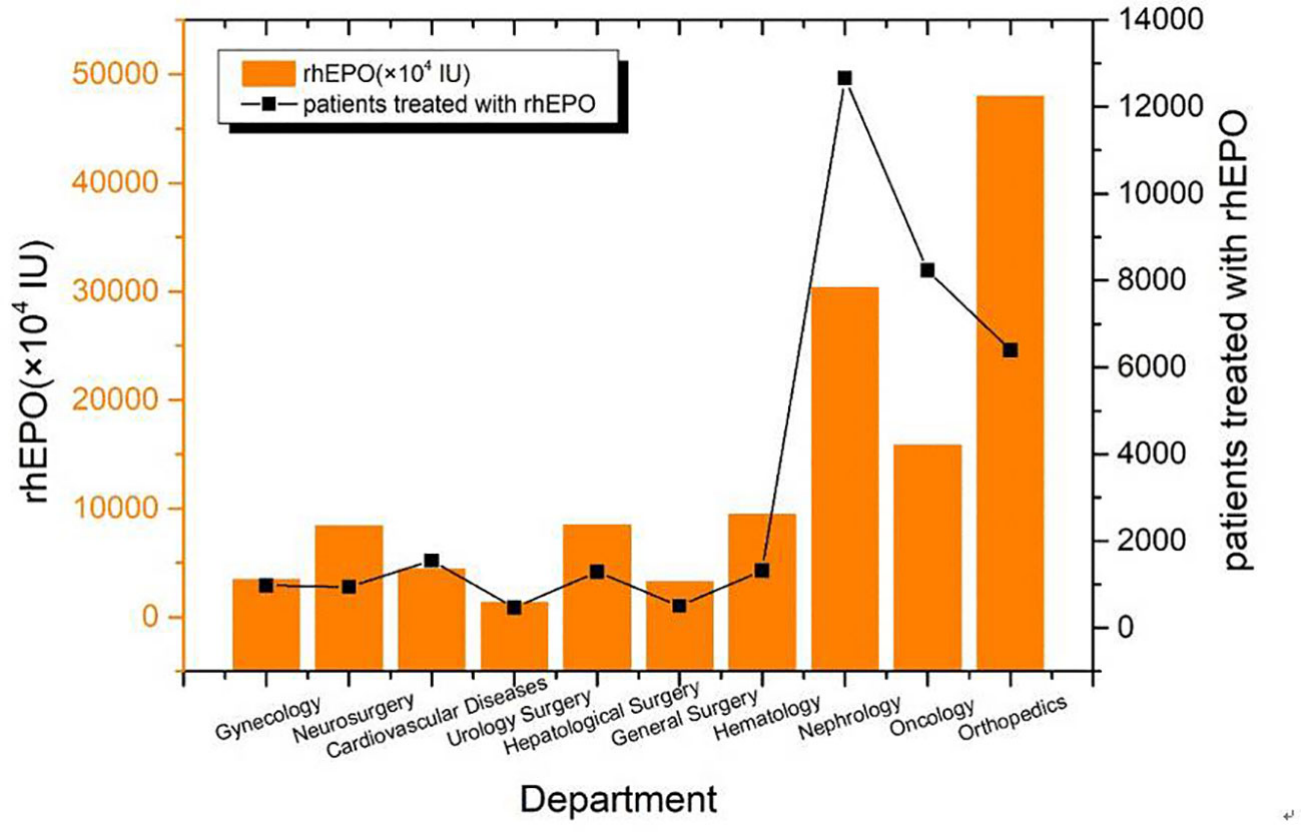
The total amount of rhEPO used by the main departments from 2009 to 2018.

The total amount of drugs used in the main drug departments is 132861×10^4^ IU and the total number of patients is 34326. The top three departments were orthopedics, nephrology and oncology. The average doses were (5.83 ± 4.85) × 10^4^ IU, (2.76 ± 2.09) × 10^4^IU and (6.4 ± 4.99) × 10^4^IU, respectively (**Figure 3**).

### Disease Distribution and Therapeutic Effect Evaluation of Drug Patients

In the medical records of 35829 patients, 5677 cases (15.8%) had incomplete medical records and 30152 cases (84.2%) had complete medical records before and after treatment. The curative effect was evaluated according to the results of laboratory Hb and Hct before and after treatment in hospital (**Table 3**).

**Table 3:** Disease Distribution and Therapeutic effect of Drug patients.

Indication	Cases	Proportion, %	Efficiency, %	Hb/(g·L^−1^)			*P*	Hct/%		*P*
				Before	After			Before	After	
**Indications**	20266	67.2	48.8	88.84 ± 20.53	101.4 ± 18.39	<0.05		0.27 ± 0.06	0.30 ± 0.05	<0.05
Anemia caused by renal failure	10896	36.1	43.5	83.96 ± 17.50	95.47 ± 14.49	<0.05		0.25 ± 0.05	0.29 ± 0.04	<0.05
Dialysis	967	3.2	43.1	83.04 ± 18.63	93.97 ± 14.77	<0.05		0.25 ± 0.05	0.28 ± 0.05	<0.05
Non-Dialysis	9929	32.9	43.6	84.05 ± 17.39	95.62 ± 14.46	<0.05		0.25 ± 0.05	0.29 ± 0.04	<0.05
Anemia caused by chemotherapy for non-bone marrow malignant tumors	3040	10.1	40.2	86.73 ± 0.05	97.11 ± 16.78	<0.05		0.26 ± 0.05	0.29 ± 0.05	<0.05
Perioperative erythrocyte mobilization	6330	20.9	–	97.38 ± 23.46	112.52 ± 0.06	<0.05		0.29 ± 0.07	0.34 ± 0.06	<0.05
**Off-label**	9886	32.8	50.7	87.21 ± 22.56	105.75 ± 20.54	<0.05		0.26 ± 0.07	0.32 ± 0.06	<0.05
Tumor associated anemia (non-chemotherapy)	8003	26.5	62.1	89.86 ± 22.61	107.18 ± 20.48	<0.05		0.27 ± 0.07	0.34 ± 0.70	<0.05
Chronic disease anemia	1653	5.5	74.0	76.02 ± 18.29	99.70 ± 19.34	<0.05		0.23 ± 0.06	0.30 ± 0.06	<0.05
Hemorrhagic anemia	150	0.5	73.3	73.1 ± 19.43	95.69 ± 18.11	<0.05		0.22 ± 0.06	0.29 ± 0.05	<0.05
Neonatal anemia	23	0.08	73.9	97.22 ± 20.20	134.39 ± 25.99	<0.05		0.29 ± 0.06	0.40 ± 0.08	<0.05
Other	57	0.2	66.7	78.38 ± 11.83	83.13 ± 14.59	<0.05		0.24 ± 0.04	0.25 ± 0.05	<0.05
**Total**	30152	100	53.1	88.36 ± 21.31	102.96 ± 19.28	<0.05		0.26 ± 0.06	0.31 ± 0.06	<0.05

### Hematopoietic Effect of rhEPO

There were significant differences in MCV (mean cell (RBC) volume), RDW (Red cell distribution width), MCHC (mean cell (RBC) Hb concentration) and reticulocyte percentage before and after rhEPO treatment for patients with indications. T values were -8.1997,-21.3912,-3.2073,-12.8409, respectively (P<0.05) (**Table 4**).

**Table 4:** Laboratory detection of hematopoiesis in indications patients treated with rhEPO.

Observation time		Anemia caused by renal failure	*P*	Anemia caused by chemotherapy for non-bone marrow malignant tumors	*P*	Perioperative erythrocyte mobilization	*P*
MCV	Before	89.74 ± 6.11	<0.05	89.39 ± 7.88	<0.05	89.29 ± 2.49	<0.05
	After	90.11 ± 5.88		89.98 ± 7.31		89.87 ± 6.67	
RDW	Before	14.01 ± 1.82	<0.05	15.44 ± 2.80	<0.05	15.04 ± 2.49	<0.05
	After	14.43 ± 2.01		15.91 ± 3.01		15.39 ± 2.56	
MCHC	Before	331.9 ± 13.8	<0.05	331.2 ± 18.25	>0.05	333.5 ± 14.7	>0.05
	After	332 ± 13.25		331.9 ± 14.9		333.5 ± 13.7	
Reticulocyte percentage	Before	2.28 ± 1.54	<0.05	2.25 ± 2.15	<0.05	2.21 ± 1.79	>0.05
	After	2.74 ± 1.65		2.44 ± 2.07		2.35 ± 1.93	

### Effect of rhEPO on RBCs Transfusion

From January 2009 to December 2018, the patients of anemia caused by renal failure, anemia caused by chemotherapy for non-bone marrow malignant tumors, RBCs mobilization during perioperative period, according to whether or not to use rhEPO at the same time, they were divided into two groups (medication group and non-medication group) to compare RBCs transfusion volume. rhEPO medication can significantly reduce RBCs transfusion in patients with anemia caused by renal failure and anemia caused by chemotherapy for non-bone marrow malignant tumors. The volume of blood transfusion in patients with RBCs mobilization in perioperative period was significantly higher than that in patients non-medication. The postoperative Hb was (115.50 ± 21.07) g/L in the medication group and (122.58 ± 21.59) g/L in the non- medication group (**Table 5**).

**Table 5:** Effect of rhEPO on transfusion.

Indication	Total	Average RBCs transfusion volume (U)	Medication			Non-medication		Z P
			cases	Average RBCs transfusion volume (U)		cases	Average RBCs transfusion volume (U)	
Anemia caused by renal failure	5,441	2.13 ± 0.75	1,690	1.94 ± 0.55		3751	2.29 ± 0.79	−13.93 0.00
Anemia caused by chemotherapy for non-bone marrow malignant tumors	7,067	2.56 ± 0.97	1,140	2.41 ± 0.82		5927	2.68 ± 0.98	−2.65 0.00
Perioperative erythrocyte mobilization	41,408	5.46 ± 5.08	6,251	6.75 ± 6.00		35157	5.21 ± 4.79	25.08 0.00

### Irregular Use of rhEPO

35829 of the patients treated with rhEPO, 16.4% of the patients were not tested for Hb and Hct before treatment; In addition to subcutaneous injection and intravenous administration, intramuscular injection and oral irregular administration accounted for 0.4%; 11.8% of patients with perioperative erythrocyte mobilization ≥ 130g/L and 1.7% of patients with Hb ≥ 150g/L at the end of medication. The timing of administration of anemia caused by renal failure was ≤ 120g/L, which was unreasonable (1.7%), The initial value of administration of anemia caused by chemotherapy for non-bone marrow malignant tumors should be ≤ 120g/L, which is unreasonable (3.2%) and contraindication (0.3%) (**Table 6**).

**Table 6:** The main use of drugs and unreasonable.

Content	cases/total	Incidence rate %
Wrong way of medication.	138/35,829	0.4
The laboratory examination before medication is incomplete.	2551/35,829	7.1
Errors in the drugs medication for RBCs mobilization in perioperative patients.	745/6,330	11.8
Hb exceeds 150 g/L after therapy of medication in perioperative patients.	110/6330	1.7
Unreasonable timing of medication of anemia caused by renal failure. Unreasonable timing of medication of anemia caused by chemotherapy for non-bone marrow malignant tumors.	181/10,896 97/3,040	1.7 3.2
Contraindication (hypertension) medication.	64/35,829	0.2

## Discussion

RhEPO is used globally in the treatment of chronic nephropathy and anemia caused by chronic kidney disease(Cowper et al., 2018). FDA recommends the application of chronic renal failure, malignant tumor chemotherapy, zidovudine treatment of AIDS infection, elective surgery patients (Bilenker et al., 2002). There are differences in the scope of application recommended by FDA and CFDA (**Table 1**). This survey collected data on all patients using rhEPO in a single center of the largest medical institution in North China from January 2009 to December 2018. The majority of drug users are young people (71.1%) (**Table 2**). Compared with 2009, the number of rhEPO users in 2018 increased by 403%, and the usage of rhEPO increased by 536% (**Figure 1**). In the survey, the highest proportion of drug use was anemia caused by renal failure, followed by preoperative erythrocyte mobilization and anemia caused by chemotherapy for non-bone marrow malignant tumors (**Figure 2**). Joshua H(Bilenker et al., 2002) reported that tumor-related diseases accounted for more than 50% of the usage, followed by kidney disease and surgery among patients who used rhEPO.

In recent years, the non-hematopoietic effect of rhEPO has also been paid close attention to, and it has been applied in many fields, such as clinical nervous system, retinal diseases and so on(Ehrenreich et al., 2011). The results of this study show that orthopedics, neurosurgery, cardiovascular disease, hepatobiliary surgery and general surgery are widely used (**Figure 3**). In our study, patients with off-label accounted for 32.9% (**Table 3**), and less than 51% of the University of Pennsylvania Hospital survey(Bilenker et al., 2002).

The study of rhEPO in the treatment of tumor-associated anemia (CRA) has been reported in recent years. It is certain that rhEPO can effectively correct CRA. The main causes of CRA include chemotherapy, tumor surgery, the tumor itself, and tumor-related malnutrition (Beutel and Ganser, 2007; Nguyen et al., 2013; Consensus expert Group on diagnosis and treatment of Renal anemia, N. B. o. C. M. A, 2018). In our study, the proportion of patients with tumor-associated anemia caused by chemotherapy and non-chemotherapy accounted for 36.6% (**Table 3**), which was consistent with 33% reported by the University of Pennsylvania Hospital (Bilenker et al., 2002). There was a significant difference in the level of Hb in CRA patients treated with rhEPO before and after treatment. According to the guidelines for Clinical practice of CRA in China (Consensus expert Group on diagnosis and treatment of Renal anemia, N. B. o. C. M. A, 2018) and rhEPO product specification, rhEPO is definitely not recommended for patients with non-chemotherapeutic CRA, However, it was found that the effective rate of rhEPO in patients with non-chemotherapeutic tumor-associated anemia was higher (62.1%), even higher than that of tumor-associated anemia after chemotherapy (40.2%) (p <0.05) (**Table 3**). In recent years, EPO treatment is also recommended for patients with malignant tumors of the hematopoietic system, palliative treatment, and tumor-associated inflammation (Ma et al., 2016). In practice, due to the complex condition of tumor patients, the scope of use of rhEPO still needs to be explored. It is suggested that when the scope of rhEPO indications is updated, the consideration of rhEPO use for non-chemotherapeutic patients with tumor-associated anemia should be included in the extended scope of recommended indications.

Among the other off-label, rhEPO was mainly used in chronic disease anemia (5.5%) (**Table 3**), which is a secondary anemia caused by chronic infection, chronic inflammation, malignant tumor and trauma for 1-2 months (Fujiwara et al., 2013). It has been reported that there may be insufficient secretion of EPO in patients with chronic disease anemia. Supplementation of EPO can promote bone marrow proliferation, prevent apoptosis of protoerythrocytes and increase the total number of erythrocytes(Wan et al., 2014). But it still needs more evidence-based medical evidence to support it. For the diseases used in off-label, the primary diseases should be treated first, and rhEPO should not be used directly to avoid the occurrence of adverse events. Gobert S et al. reported that infection and inflammation are one of the factors leading to rhEPO resistance (Gobert et al., 1995). It is obvious that the direct use of rhEPO in chronic infectious inflammatory anemia may be unreasonable.

51.2% of the patients did not respond to the indications recommended by CFDA (Hb increased<10g/L) (**Table 3**). EPO resistance may be the main cause of ineffective treatment. With regard to the concept of EPO resistance, according to the clinical practice guidelines for anemia caused by renal failure issued by the National Kidney Disease Foundation (NKF), after using rhEPO 400 IU/kg or 20,000 IU every week for three months, If the blood hemoglobin concentration of the patient is still less than 11g/dL, it can be diagnosed as rhEPO resistance (KDOQI and National Kidney Foundation, 2006). According to the consensus of Chinese kidney disease experts, after subcutaneous injection of rhEPO reached weekly 300 IU/kg or intravenous injection of 500IU/kg for four months, it still could not reach or maintain the target value, which was called EPO resistance (Consensus expert Group on diagnosis and treatment of Renal anemia, N. B. o. C. M. A, 2018). There are many known reasons for resistance, of which iron deficiency is the main factor, followed by infection, malnutrition, secondary hyperparathyropathy, and EPO resistance can also be induced by autoimmune diseases, aluminum poisoning and angiotensin converting enzyme inhibitors (Jacobs et al., 1985; Freyssinier et al., 1999; Kashii et al., 2000; Bouscary et al., 2003; Bohlius et al., 2005). The use of rhEPO can also stimulate the body to produce EPO antibodies. leading to EPO resistance. In this study, no clinical detection of blood EPO level and related antibodies were found., and no large sample data on EPO resistance were found in China. Clinical attention has not been paid to the detection of EPO level and related antibodies. It is suggested that the curative effect should be evaluated in time during the treatment of rhEPO, the occurrence of EPO resistance should be paid more attention to, the individualized treatment plan should be adjusted and formulated at any time.

Bohlius et al. reported that the use of EPO could significantly improve the level of Hb, and reduce the demand for allogenic blood transfusion (Bohlius et al., 2005). In this study, the RBC(s) transfusion in patients with rhEPO indication/non-medication was analyzed, and the results showed that the use of rhEPO in patients with anemia caused by renal failure and anemia caused by chemotherapy for non-bone marrow malignant tumors could significantly reduce RBCs transfusion. However, for patients with RBCs mobilization in perioperative period, the RBCs transfusion volume in the non-medication group was lower than that in the medication group (**Table 5**). We infer that this is due to the fact that the restrictive RBC(s) transfusion strategy has not been strictly implemented, and the effect of EPO on saving RBC(s) is not reflected due to over-indications RBC(s) transfusion. In addition, the monitoring of hematopoiesis-related laboratory indexes in the study showed that the use of rhEPO had a significant effect on hematopoiesis in patients (**Table 4**).

In this study, 7.1% of the patients did not detect the levels of Hb and Hct before using rhEPO. In the mode of administration, intramuscular injection, which is not standardized, still accounts for 0.4% (**Table 6**).

There are two ways of administration of rhEPO: intravenous injection and subcutaneous injection. After intravenous administration, rhEPO reaches the plasma peak concentration fastest, but the half-life is short (about 5 to 10 hours). Subcutaneous injection has a long half-life (about 12 to 18 hours), so it is often recommended during perioperative period (Bohlius et al., 2009). In addition, it was noted that in 11.8% of the patients with perioperative RBC(s) mobilization, the preoperative Hb was greater than 130g/L, and 1.7% of the patients were lack of Hb and Hct monitoring during medication, and did not stop medication in time, resulting in Hb greater than 150g/L at the end of medication. For patients whose hemoglobin is higher than 130g/L before operation, the standard of WHO anemia has not been met, the use of rhEPO will improve the blood viscosity and increase the risk of thromboembolism. When Hb reaches 140g/L (Garrido-Martín et al., 2012) and 150g/L (Colomina et al., 2004) in the process of using rhEPO, rhEPO should be stopped in time, and blood coagulation indexes should be closely monitored to prevent the occurrence of various forms of thromboembolism. These two conditions of RBCs mobilization in perioperative period do not meet the requirements of drug use and belong to unreasonable drug use. According to the expert consensus on the diagnosis and treatment of anemia caused by renal failure (2014) and the expert consensus on the rational use of Recombinant Human Erythropoietin in anemia caused by renal failure (2007), the timing of medication for anemia caused by renal failure and anemia caused by chemotherapy for non-bone marrow malignant tumors should be Hb≤ 120g/L. In this study, 1.7% of patients with anemia caused by renal failure and 3.2% of patients with anemia caused by chemotherapy for non-bone marrow malignant tumors did not meet the requirements of the guidelines. Hypertension is the most common adverse drug reaction in rhEPO, the hypertension caused by rhEPO is mainly related to the rapid increase of hemoglobin after the improvement of anemia, which leads to the increase of blood viscosity, vascular tension and peripheral small vessel resistance. Therefore, routine blood pressure monitoring is recommended for patients who use rhEPO. However, mild elevated blood pressure may be a sign of improvement in anemia. The Organization for improving the prognosis of Global Kidney Disease recommends that EPO treatment should not be stopped or interrupted because of high blood pressure (Drüeke and Parfrey, 2012). If it is clear that hypertension caused by rhEPO can be treated with antihypertensive drugs. In this study, it was found that 64 patients with hypertension were treated with rhEPO, which was contraindication, but the blood pressure was well controlled and did not stop after antihypertensive drug treatment.

In summary, the investigation on the clinical application of rhEPO in the largest medical institution in North China shows that the amount of rhEPO use has been increasing continuously in recent years. However, there was still a certain proportion of patients with rhEPO indications (renal anemia) who did not use rhEPO, but were transfused RBC(s) to correct anemia. About 1/3 rhEPO is used Off-label approved by CFDA, but its curative effect is similar to that of the recommended indications, suggesting that with the accumulation of evidence-based medicine data in clinical application, the scope of approved application can be expanded at the right time. At the same time, the unreasonable use of rhEPO and RBC(s) transfusion Off-label still exists, and more attention should be paid in clinic. It is suggested that clinicians should pay attention to observe the curative effect of drugs in practical application and adjust the scope of application properly on the basis of using drugs according to the standard of indications. Pay attention to the decision-making of medication and blood transfusion in the treatment of anemia. However, there are some limitations in the study, which can only show that the research results of a single center cannot represent the situation of multi-centers in the country. But the first Medicine Center of the Chinese PLA General Hospital is the largest single center of medical institutions in North China.The annual admission of more than 200000 patients from all over the country, with the basic advantages of big data research, the results are representative. In the future, researchers in medical institutions should further design multicenter research programs to fully understand the clinical application of rhEPO in China.

## Data Availability Statement

All datasets generated for this study are included in the article/supplementary material.

## Ethics Statement

This study has been approved by Medical Ethics Committee of the General Hospital of the Chinese people’s Liberation Army.(code:S2019-290-01).

## Author Contributions 

X-ZG, L-LW, YY, and D-QW participated in research design. X-ZG, XP, YY, LL, X-LS, and X-JZ performed the experiments and(or) data analysis. X-ZG and YY contributed to the writing of the manuscript.

## Conflict of Interest

The authors declare that the research was conducted in the absence of any commercial or financial relationships that could be construed as a potential conflict of interest.
